# Prevalence of Anxiety and Depression Among Children and Adolescents in Low‐ and Middle‐Income Countries—A Systematic Review

**DOI:** 10.1176/appi.prcp.20250026

**Published:** 2025-07-31

**Authors:** Karishma Ramdhonee‐Dowlot, Kieran Balloo, Evren Morgül, Cecilia A. Essau

**Affiliations:** ^1^ School of Psychology University of Roehampton London UK; ^2^ SCU College Southern Cross University Gold Coast Queensland Australia; ^3^ Surrey Institute of Education University of Surrey Guildford UK

## Abstract

**Background:**

This study reviews literature examining the prevalence of emotional problems (i.e., anxiety and depressive symptoms) among child and adolescent populations living in low‐ and middle‐income countries (LMICs).

**Method:**

Following recommendations of the Preferred Reporting Items for Systematic Reviews and Meta‐Analyses (PRISMA) statement, PsycINFO (EBSCO), and MEDLINE (PubMed) databases were searched for articles published from January 1, 2000 to April 30, 2024. Quantitative studies reporting epidemiological/prevalence data were included in the review.

**Results:**

Fifty‐one studies (*N* = 208,842) across 23 LMICs were included in a qualitative synthesis. Measures used in studies on LMIC samples were comparable to those used in high‐income countries (HICs). The prevalence of emotional problems among children and adolescents in LMICs appears to be higher than in HICs. The synthesis showed an estimated prevalence ranging from 1% to 58% for depression, 1% to 30% for anxiety, and 1% to 41% for overall emotional problems (i.e., both anxiety and depression). The review indicates that types of anxiety problems with the highest prevalence estimates related to symptoms of generalized anxiety (18.2%), separation anxiety (14.0%), posttraumatic stress (32.0%), specific phobia (20.9%), and social phobia/social anxiety (20.2%).

**Conclusions:**

There are a number of potential risk factors for child and adolescent populations living in LMICs, which could explain the elevated prevalence: an increased vulnerability related to being left‐behind by one or both migrant parents, orphaned status due to high rates of mortality from poor health conditions, experiences of war conflict and violence, childhood maltreatment, and poverty. These findings highlight the urgent need for scalable, culturally sensitive mental health screening, prevention, and intervention programs tailored to LMIC contexts. They also have important implications for global child and adolescent mental health policy, funding priorities, and capacity‐building efforts.

## Highlights

- High variability has been found between high‐income countries (HICs) and low‐ and middle‐income countries (LMICs) in the prevalence rates of anxiety and depression in child and adolescent populations.
- The prevalence of emotional problems in LMICs seems to be higher than in HICs.
- A systematic review is essential for acquiring accurate prevalence rates that could inform research and intervention priorities in the child and adolescent population of LMICs.
- These results are essential to service, training, and research planning in LMICs and should be used in a manner that will promote sound and sustainable anxiety and depression prevention programmes for young people in LMICs.

Emotional problems, such as anxiety and depression, are the most common mental health problems experienced during childhood and adolescence (1). Recent studies continue to highlight the significant burden of these disorders, with global prevalence rates indicating a steady increase over the past decade (2). Lifetime prevalence of mental disorders for adolescents in the United States has been found to be as high as 31.9% for anxiety disorders, and 11.7% for depression (3). By 2030, depression alone is projected to be the third leading cause of disease burden in low‐income countries and the second highest cause of disease burden in middle‐income countries (4). A recent study highlights that a considerable number of adolescents in 26 low‐ and middle‐income countries (LMICs) show daily signs of anxiety and depression, with higher prevalence among those with functional difficulties (5). Yatham et al. (6) obtained prevalence estimates for youth in LMICs ranging up to 27% for anxiety symptoms, up to 28% for depressive symptoms, and for posttraumatic stress disorder (PTSD), the estimate ranged as high as 87% in adolescents who had experienced traumatic events.

When comparing the prevalence rates of children and adolescents in high‐income countries (HICs) to those in LMICs, a large variability has often been found which has largely been attributed to methodological limitations concerning the nature of the population tested and the diagnostic limitations. These methodological issues include variability in sampling strategies (e.g., school‐based vs. household samples), differences in age ranges assessed, and inconsistent use of validated diagnostic instruments. The majority of these reviews have focused on children and adolescents experiencing adverse events such as war, conflict and/or humanitarian crises (6, 7, 8, 9). Some reviews have focused on those who fit into clinical diagnostic categories (10), despite the presence of many more children and adolescents who still have impaired levels of psychological distress but who remain undiagnosed (11). For example, many children and adolescents in LMICs struggle with emotional problems but they do not form a discrete group. Furthermore, LMICs lack the human resources to carry out assessments, and misdiagnosis may occur due to the use of unreliable and invalid assessment tools (12, 13). This masks symptoms and the presence of comorbid disorders. In addition, cross‐cultural differences in how emotional problems are expressed and perceived may affect the interpretation of standardized tools developed in high‐income contexts. Misdiagnosis and under‐detection of symptoms often lead to young people not being given appropriate, or even any, intervention. This often leads to worsening of emotional problems, such as the development of chronic depression, self‐harm behaviors, or school refusal over time. Hence, the frequency of emotional problems such as anxiety and depression among the child and adolescent population in LMICs raises questions about the real nature and extent of emotional problems experienced by children and adolescents in LMICs.

Brief screening tools are essential for mental health care in LMICs. LMICs face challenges related to stigma and labeling of emotional disorders, and low human resources and service facilities (13). Mental health professionals have time and training constraints to administer complex diagnostic interviews to all individuals at risk of mental illness. Adopting appropriate screening instruments can also enhance research and training in detecting emotional problems in LMICs. By providing a succinct overview of symptoms of emotional problems, the screening instruments train mental health workers on what to look for and thus improve their ability to detect emotional problems. Evidence suggests that screening tools are more likely to perform well in a given setting, and it should be enhanced by local validation wherever possible (12).

There is an urgent need for early detection in order to inform effective prevention strategies for emotional problems in LMICs. However, research into child and adolescent mental health policy in LMICs receives insufficient attention in the academic arena, which reduces public awareness and limits the roles of experience and expertise in policymaking (13). Recent findings emphasize the critical gap in mental health infrastructure and the need for integrated community‐based interventions to address the growing burden of emotional problems among young people (14). As discussed in a recent systematic review (15), efforts to tackle the mental health needs of children and adolescents remain a neglected issue in LMICs, with less than 10% of all randomized controlled trials on school‐ or community‐based psychological interventions conducted in LMICs. One of the reasons for this low percentage is that delivering targeted interventions may be challenging as they tend to be resource intensive (15). One of the major challenges remaining relates to a lack of data and evidence on child and adolescent mental health, from service statistics to programme evaluation (1, 13). To better solve challenges for LMICs, more research based on local experience and expertise is in extreme need.

Accurate prevalence estimates in LMICs are essential to inform service planning, public policies, and the provision of care (1). Moreover, the identification of prevalence estimates can contribute to addressing questions about etiology and inform the design of future studies in LMICs (16). To date, there is a lack of research in LMICs providing reliable prevalence estimates of emotional problems among children and adolescents, which is an important requirement when determining the need for intervention. Thus, the objective of this systematic review is to provide an analysis of research on the child and adolescent population of LMICs to identify the methods for assessing, and prevalence of, emotional problems.

## METHOD

### Protocol and Registration

This review was developed in accordance with the recommendations of the Preferred Reporting Items for Systematic Reviews and Meta‐Analyses (PRISMA) statement (17). The protocol for this review was registered in the International Prospective Register of Systematic Reviews (PROSPERO) database prior to data extraction (CRD42018109406).

### Information Sources and Search Strategy

PsycINFO (EBSCO) and MEDLINE (PubMed) databases were searched for articles published from January 1, 2000 to April 30, 2024. A comprehensive search using both keywords (also free text searching) and subject‐headings (also controlled vocabulary searching) included: (a) “child”/”adolescent” terms (e.g., child, adolescent, youth, minors); (b) “emotional problems”/“mental health problems” terms (e.g., anxiety, depression, internalizing); (c) “low‐ and middle‐income country”/“developing country” terms (e.g., [underdeveloped, third world, underserved] country, population, nation); and (d) “study design” terms (e.g., epidemiological, cross‐sectional, cohort). The master search strategies used for searching PsycINFO and MEDLINE are provided in online supplement. Reference lists of all included articles were then scanned to identify any relevant articles that may have been missed in the online search. Eligibility criteria are elucidated in Table 1.

**TABLE 1:** Eligibility criteria.

Criteria	Specifications
Participants	Children and adolescents aged 0 to 19 years of age (based on the WHO categorization of childhood and adolescence), being assessed for emotional problems, mental health problems and/or issues related to anxiety and/or depression symptoms
Settings	Participants recruited from community settings, educational contexts or birth cohorts, in LMICs. Designation as an LMIC was based on the World Bank categorization of countries (https://www.worldbank.org) in 2018^a^
Types of assessment and outcomes	Diagnostic interviews or validated questionnaire measures that provide cut‐off scores based on either DSM or ICD symptoms criteria, or validated measures of clinical level of anxiety and/or depressive related symptoms. Diagnosis of anxiety or depression was not a basis for inclusion or exclusion. Instead, studies were considered eligible if they included standardized outcome measures or subscales from a composite measure. Studies with multiple targeted research outcomes were also included if they had one outcome defined as measuring the prevalence of anxiety and depression related symptoms
Study type	Quantitative, such as observational, cohort and intervention methods, reporting epidemiological/prevalence data
Exclusion criteria	Studies conducted in HICs; studies conducted on clinical samples (e.g., inpatients in hospitals); special samples (e.g., those who experienced special events such as earthquakes, or living in special settings, such as an orphanage or dormitory); participants with intellectual disabilities or undergoing treatment for any health condition; studies that did not separate data for children and adolescents from the data of older participants; single case or case series methodologies; and/or studies reporting only qualitative information. Articles that were not peer‐reviewed or reported in the English language were also excluded. If there was more than one paper reporting data from the same sample, only the study reporting the findings most closely related to the study criteria was included

a World Bank classifications are updated every year. For the current study, the year 2018 was selected, as this is the year that the initial systematic review protocol was developed.

### Study Selection

3Eligibility assessment (title/abstract screening and full‐text assessment) of identified articles was initially performed by the lead author, with an additional updated search performed by the third author. An independent reviewer supported the review process. This reviewer independently assessed a random sample of the included full‐text articles (approximately 20%) to verify that the inclusion and exclusion criteria were applied consistently. In cases where there was uncertainty about a study's eligibility, the independent reviewer was consulted, and disagreements were resolved through discussion among the authors. No formal inter‐rater reliability was calculated, but consistency in judgment was maintained through frequent consultation.

Abstracts that were clearly irrelevant for the current study were discarded, while the remaining full‐texts were reviewed for their applicability to the inclusion and exclusion criteria. Where the author was unsure whether a paper met the eligibility criteria, the independent reviewer was consulted. The independent reviewer further assessed a sample of the final included articles to ensure that they met the inclusion/exclusion criteria. Disagreements, if present, were resolved by consensus.

### Data Extraction

The following information was extracted from studies included in the review: study characteristics (author, publication date, country, sample size); sample recruitment (e.g., community, cohort, school); participant characteristics (age group, percentage of boys and girls, if data were available); assessment characteristics (diagnostic measures, standardized questionnaires) and prevalence rate of all types of anxiety and depression disorders (percentage of girls, boys, and/or both).

### Data Synthesis

The large heterogeneity between populations and methods did not allow for a meaningful meta‐analysis of results. Therefore, what is presented below is a qualitative synthesis of prevalence data. Prevalence rates were extracted directly as reported in each included study. No new calculations, weighting, or statistical pooling were performed. Where studies reported multiple prevalence estimates (e.g., by symptom severity or diagnostic subtype), the most inclusive prevalence figure (e.g., “any anxiety disorder” or “any depressive disorder”) was used to represent the overall estimate. To provide an overview of the variation across studies, minimum and maximum values were reported for each type of emotional problem.

## RESULTS

### Selection of Studies

**FIGURE 1:**
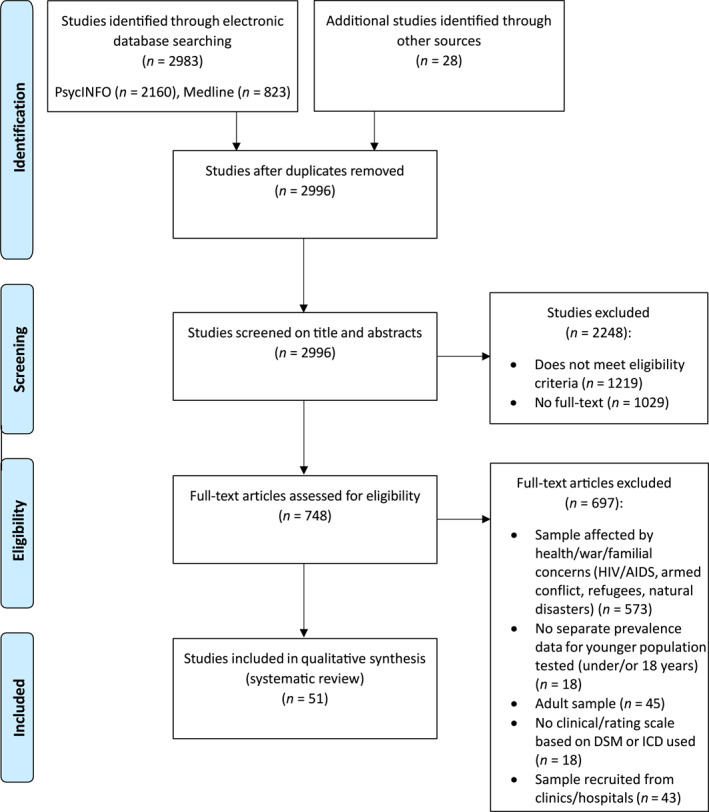
Preferred Reporting Items for Systematic Reviews and Meta‐Analyses (PRISMA) flow diagram of study selection.

A total of 2996 abstracts were identified through the searches of PsycINFO and MEDLINE, and other sources such as reference lists and forum posts (*n* = 28). Following removal of duplicates and abstract screening, and full‐text screening, 51 studies met the eligibility and inclusion criteria and were retained for synthesis. A flow diagram outlining study selection is presented in Figure 1. Full statistics related to the characteristics of each of these studies can be found in online supplement. A summary of these characteristics follows below. Participants across studies ranged in age from 1 to 20 years, with the majority of prevalence estimates derived from school‐aged children and adolescents (approximately 10–18 years). Detailed age ranges are presented in Table 2.

**TABLE 2:** Full characteristics of studies included in review.

	Author (year)	Country	Design	*N*	Age range (years)	Gender		Instrument/s	Prevalence rate (%)		
						Male (%)	Female (%)		Male (%)	Female (%)	Both (%)
Anxiety symptoms (four studies)											
1.	Abbo et al. (18)	Uganda	CS Multistage sampling of children in four rural districts	1587	3–19	46.3	53.7	MINI International Neuropsychiatric Interview for Children and Adolescents (MINI‐KID) DSM‐IV oriented	Any anxiety disorder: 23.1% Generalized anxiety disorder: 0.54% Posttraumatic stress disorder: 6.3% Obsessive compulsive disorder: 0.41% Separation anxiety disorder: 5.4% Social phobia (social anxiety disorder): 4.9% Panic disorder (with agoraphobia): 2.0% (3.4%) Specific phobia: 13.2%	Any anxiety disorder: 29.7% Generalized anxiety disorder: 2.1% Posttraumatic stress disorder: 6.9% Obsessive compulsive disorder: 0.70% Separation anxiety disorder: 6.1% Social phobia (social anxiety disorder): 5.5% Panic disorder (with agoraphobia): 3.9% (4.1%) Specific phobia: 17.9%	Any anxiety disorder: 26.6% Generalized anxiety disorder: 1.4% Posttraumatic stress disorder: 6.6% Obsessive compulsive disorder: 0.57% Separation anxiety disorder: 5.8% Social phobia (social anxiety disorder): 5.2% Panic disorder (with agoraphobia): 3.0% (3.8%) Specific phobia: 15.8%
2.	Adewuya et al. (19)	Nigeria (Western)	CS Multistage sampling of secondary school students	1090	13–18	56.4	43.6	MINI International Neuropsychiatric Interview for Children and Adolescents (MINI‐KID) DSM‐IV oriented	A 12‐month diagnosis of at least one DSM‐IV anxiety disorder: 11.4% Panic disorder (with or without agoraphobia): 1.8% Separation anxiety disorder: 2.1% Generalized anxiety disorder: 2.8% Specific anxiety disorder: 2.0% Obsessive compulsive disorder: 1.5% Posttraumatic stress disorder: 0.0% Social phobia (social anxiety disorder): 3.6%	A 12‐month diagnosis of at least one DSM‐IV anxiety disorder: 19.6% Panic disorder (with or without agoraphobia): 3.2% Separation anxiety disorder: 2.1% Generalized anxiety disorder: 4.6% Specific anxiety disorder: 3.2% Obsessive compulsive disorder: 2.1% Posttraumatic stress disorder: 0.4% Social phobia (social anxiety disorder): 6.3%	A 12‐month diagnosis of at least one DSM‐IV anxiety disorder: 15.0% Panic disorder (with or without agoraphobia): 2.4% Separation anxiety disorder: 2.1% Generalized anxiety disorder: 3.6% Specific anxiety disorder: 2.5% Obsessive compulsive disorder: 1.7% Posttraumatic stress disorder: 0.2% Social phobia (social anxiety disorder): 4.8%
3.	Demir et al. (20)	Turkey	CS Two‐stage cluster sampling of students between grade 4 to grade 8 from 3 schools	1482	9–16	49.8	50.2	Stage 1: The Social Anxiety Scale for Children‐Revised (SASC‐R) and the Capa Social Phobia Scale for Children and Adolescents (CSPSCA) Stage 2: The Schedule for Affective Disorders and Schizophrenia for School‐Age Children‐Present and Lifetime Version (K‐SADS‐PL) DSM‐IV oriented	Social anxiety disorder: 4th–5th graders: 1.8% 6th–8th graders: 3.2%	Social anxiety disorder: 4th–5th graders: 3.5% 6th–8th graders: 6.4%	Social anxiety disorder: 3.9%
4.	Nair et al. (21)	India	CS A representative community sampling of adolescents from Kerala, southern India	500	11–19	36.6	63.4	The Screen for Child Anxiety Related Emotional Disorders (SCARED) questionnaire DSM‐IV‐TR oriented	(Based on SCARED Indian Cutoff of ≥21) All anxiety disorders = 6.6% Panic disorder = 7.4% Generalized anxiety disorders = 5.2% Separation anxiety disorder = 1.4% Social anxiety disorder = 5.6%	(Based on SCARED Indian Cutoff of ≥21) All anxiety disorders = 19.2% Panic disorder = 16% Generalized anxiety disorders = 13% Separation anxiety disorder = 7.6% Social anxiety disorder = 14.6%	(Based on SCARED Indian Cutoff of ≥21) All anxiety disorders = 25.8% Panic disorder = 23.4% Generalized anxiety disorders = 18.2% Separation anxiety disorder = 9.0% Social anxiety disorder = 20.2%
Depressive symptoms (25 studies)											
5.	Adeniyi et al. (22)	Nigeria	CS A stratified two‐stage sampling of adolescents from 11 secondary schools (both public and private)	1100	12–17	48.9	51.1	The Children's Depression Inventory (CDI) DSM‐IV oriented	‐	‐	Mild to moderate depression (CDI scores of 1–19) = 23.8% Definite depression (CDI scores of ≥20) = 5.7%
6.	Adewuya et al. (23)	Nigeria	CS Multistage sampling of secondary school students from 10 schools in Western Nigeria	1095	13–18	57.7	42.3	Beck Depression Inventory (BDI) and the Schedule for Affective Disorders and Schizophrenia for School‐Aged Children—Epidemiological Version 5 (K‐SADS‐E) DSM‐IV oriented	Major depressive disorder: 5.5%	Major depressive disorder: 8.9%	Major depressive disorder: 6.9%
7.	Hecker et al. (24)	Tanzania	CS Random sampling of primary school children from grades 2 to 7	409	6–15	52.0	48.0	Children's Depression Inventory (CDI) DSM‐IV oriented			Depression (CDI scores cut‐off point of ≥12) = 14%
8.	Demir et al. (25)	Turkey	CS Two‐stage cluster sampling of students between grade 4 to grade 8 from 3 schools	1482	9–16	49.8	50.2	Stage 1: Children were screened using the Child Depression Inventory (CDI) Stage 2: According to the results of stage 1, 320 children were interviewed using the Schedule for Affective Disorders and Schizophrenia for School‐Age Children‐Present and Lifetime Version (K‐SADS‐PL) DSM‐IV oriented	Any depressive disorder: 3.65% Major depressive disorder: 1.35% Dysthymic disorder: 1.21% Double depression (MDD super‐imposed on dysthymia): 0.27% Depression‐not otherwise specified: 0.81%	Any depressive disorder: 4.7% Major depressive disorder: 1.74% Dysthymic disorder: 2.28% Double depression (MDD super‐imposed on dysthymia): 0.26% Depression‐not otherwise specified: 0.4%	Any depressive disorder: 4.2% Major depressive disorder: 1.55% Dysthymic disorder: 1.75% Double depression (MDD super‐imposed on dysthymia): 0.26% Depression‐not otherwise specified: 0.6%
9.	El‐Missiry et al. (26)	Egypt	CS Multistage cluster sampling of female secondary school students from 6 schools in Cairo	602	14–17	‐	100.0	The Children Depression Inventory (CDI) and the Non‐patient version of the Structured Clinical Interview for DSM‐IV axis‐I disorders (SCID‐I/NP) DSM‐IV oriented		CDI (Egyptian adolescent depression cutoff of ≥24) = 15.3% SCID‐I/NP (any depressive disorder) = 13.3% (current/point prevalence)	
10.	Fatiregun and Kumapayi (27)	Nigeria	CS Stratified cluster sampling of adolescents from 7 secondary schools in southwest Nigeria	1713	10–19	44.7	55.3	The Patient Health Questionnaire (PHQ‐9) for depression DSM‐IV oriented			Depressed (≥10 scores on PHQ‐9): 21.2% Severity of depression (based on PHQ‐9): Mild depression (5–9) = 34.7% Moderate depression (10–14) = 16.1% Moderately severe depression (15–19) = 4.6% Severe depression (≥20) = 0.5%
11.	Ding et al. (28)	China	CS Cluster sampling of children and adolescents from 5 primary and 5 secondary schools	6406	9–17	51.6	48.4	The Center for Epidemiological Studies Depression Scale (CES‐D) DSM‐IV oriented	Depressed (≥16 scores on CES‐D) = 15.1%	Depressed (≥16 scores on CES‐D) = 17.5%	Depressed (≥16 scores on CES‐D) = 16.3%
12.	Karacetin et al. (29)	Turkey	CS Multi‐center epidemiological study of a representative sample of randomly selected students of 2nd to 4th grades from 29 study centers across 8 counties in Turkey	5842	8–10	51.7	48.3	The Kiddie Schedule for Affective Disorders and Schizophrenia for School Age Children‐ Present and Lifetime Version (K‐SADS‐PL) Semi‐structured interview based on DSM‐III‐TR and DSM‐IV	Major depressive disorder (without impairment criteria) = 1.6% (with impairment criteria) = 1.0% Dysthymia (without impairment criteria) = 0.1% (with impairment criteria) = 0.1% Depressive disorder NOS (without impairment criteria) = 0.1% (with impairment criteria) = 0.1%	Major depressive disorder (without impairment criteria) = 1.9% (with impairment criteria) = 1.2% Dysthymia (without impairment criteria) = 0.1% (with impairment criteria) = 0.1% Depressive disorder NOS (without impairment criteria) = 0.1% (with impairment criteria) = 0.2%	Major depressive disorder (without impairment criteria) = 1.7% (with impairment criteria) = 1.06% Dysthymia (without impairment criteria) = 0.2% (with impairment criteria) = 0.2% Depressive disorder NOS (without impairment criteria) = 0.15% (with impairment criteria) = 0.14%
13.	Kinyanda et al. (30)	Uganda (north‐east)	CS Multistage sampling of children and adolescents from a random selection of 210 households from each county across 4 districts of northeast Uganda	1587	3–19	46.3	53.7	The MINI International Neuropsychiatric Interview for children and adolescents (MINI‐KID) DSM‐IV oriented	Any depressive disorders: 8.3% Major depressive disorder: 7.5% Dysthymia: 1.5%	Any depressive disorders: 8.8% Major depressive disorder: 7.6% Dysthymia: 2.7%	Any depressive disorders: 8.6% Major depressive disorder: 7.6% Dysthymia: 2.1%
14.	Kidman et al. (31)	Malawi	CS Cohort sample of adolescents in rural Malawi between 2008 and 2017 for children aged 10 to 16 years	2089	10–16	50.84	49.16	Beck Depression Inventory (BDI) (BDI cut‐off scores of moderate to severe depression ≥17)			Depression symptoms = 16.99%
15.	Li et al. (32)	China	CS Convenience sampling of adolescents from 2 secondary schools in Guangzhou, mainland China	1015	12–15	58.8	41.2	The Center for Epidemiological Studies‐Depression scale (CES‐D) DSM‐IV oriented	‐	‐	Mild depression (scores of CES‐D ≥ 16 and CES‐D < 21) = 17.7% Moderate depression (scores of CES‐D ≥ 21 and CES‐D <25) = 10.1% Severe depression (scores of CES‐D ≥25) = 13.4%
16.	Maurya et al. (33)	India	CS Multi‐stage sampling of adolescents and young adults in the Indian states of Uttar Pradesh and Bihar, using cohort study data	16,292	13–17	55.63	44.37	Patient Health Questionnaire (PHQ‐9) The variable was recorded as 1 “yes” meaning meeting criteria for a depressive disorder if the respondent scored five and above on 0–27 scale and 0 “no depressive symptom” if the respondent scored less than five on the scale (0–4, minimal)	Depressive symptoms = 16.6% Suicidal ideation = 2.3%	Depressive symptoms = 33% Suicidal ideation = 7.5%	
17.	Munhoz et al. (34)	Brazil (Pelotas)	CS Two‐stage cluster sampling of households in Pelotas, Brazil having children and adolescents aged 10–19 years	743	10–19	48.3	51.7	The Patient Health Questionnaire‐9 (PHQ‐9) DSM‐IV oriented	Depression (PHQ‐9 scores ≥9): 13.9%	Depression (PHQ‐9 scores ≥9): 19.8%	Depression (PHQ‐9 scores ≥9): 17.0%
18.	Nalugya‐Sserunjogi et al. (35)	Uganda	CS A representative sample of adolescents from 4 secondary schools in central Uganda	519	14–16	58.0	42.0	Children's Depression Inventory (CDI) DSM‐IV oriented	Depression (CDI scores cut‐off point of ≥19) = 17%	Depression (CDI scores cut‐off point of ≥19) = 26%	Depression (CDI scores cut‐off point of ≥19) = 21%
19.	Perera et al. (36)	Sri Lanka	CS Random sampling survey of students from 3 schools	891	14–19	49.5	50.5	The Center for Epidemiological Studies‐Depression Scale (CES‐D) DSM‐IV oriented	Depression (CES‐D cut‐off scores of ≥16) = 58.5%	Depression (CES‐D cut‐off scores of ≥16) = 56.9%	Depression (CES‐D cut‐off scores of ≥16) = 57.7%
20.	Rahman et al. (37)	Pakistan	CS Community‐based survey of all 16 to 18‐year old unmarried adolescents in one rural community in Rawalpindi District, koori Dulal	321	16–18	‐	100.0	The Structured Clinical Interview for DSM‐IV Disorders (SCID) DSM‐IV oriented		Major depressive disorder (based on SCID assessment) = 4.4%	
21.	Rodrigo et al. (38)	Sri Lanka	CS Random sampling of students from 2 random schools	445	14–18	54.4	45.6	The Center for epidemiologic studies depression scale (CES‐D) DSM‐IV oriented			Mild Depression (CES‐D scores of 16–21) = 17% Severe Depression (CES‐D cut‐off scores of ≥21) = 19%
22.	Sarkar et al. (39)	India	CS Two‐staged sampling of students of grades 1 to 7 from 4 randomly selected schools in the sub‐urban area of Kanke, Ranchi.	1851	8–12	32.9	67.1	The Schedule for Affective Disorders and Schizophrenia for School Age Children Present and Lifetime Version (K‐SADS‐PL) DSM‐IV oriented			Any depressive disorders = 3.13% Major depressive disorder = 0.81% Dysthymia = 1.51% Depressive disorder NOS = 0.81%
23.	Shaaban and Baashar (40)	Sudan	CS Two‐stage, stratified random sampling of adolescent school girls from 6 randomly selected elementary and secondary girls' schools in Khartoum, Sudan	1107	12–19	‐	100.0	The Beck Depression Inventory (BDI) DSM‐IV oriented		Overall Depression (BDI scores of 8–39) = 39.4% Moderate Depression (BDI scores of 8–15) = 28.2% Severe Depression (BDI cut‐off scores of ≥16) = 11.2% Major depressive disorder (DSM‐IV) = 4.2%	
24.	Silva et al. (41)	Brazil	Cohort follow‐up study of school children sample from Ribeirão Preto (more developed center, *n* = 790) and São Luís (less Developed center, *n* = 673) born between 1994 and 1998	1463	7–11	48.7	51.3	The Children's Depression Inventory (CDI) DSM‐IV oriented	‐	‐	Depression (CDI cut‐off scores of ≥17) weighted in Ribeirão Preto = 6.0% Depression (CDI cut‐off scores of ≥17) weighted in São Luís weighted = 21.6%
25.	Somrongthong et al. (42)	Thailand (Bangkok)	CS Survey study of adolescents living in a slum community selected through systematic random sampling of households	623	12–17	43.5	56.5	The Center for Epidemiologic Studies Depression scale (CES‐D) DSM‐IV oriented	Depression (CES‐D cut‐off scores of ≥22) = 27.4%	Depression (CES‐D cut‐off scores of ≥22) = 42.3%	Depression (CES‐D cut‐off scores of ≥22) = 36.1%
26.	Toros et al. (43)	Turkey	CS A multistage, stratified cluster sampling of randomly selected 18 secondary and high schools in Mersin, Turkey	4143	10–20	54.4	45.6	The Child Beck Depression Inventory (CBDI) DSM‐IV oriented	Depression (CBDI cut‐off scores of ≥19) = 6.6%	Depression (CBDI cut‐off scores of ≥19) = 5.9%	Depression (CBDI cut‐off scores of ≥19) = 12.6%
27.	Wang et al. (44)	China (western)	CS A multistage, stratified cluster, random sampling of children and adolescents from 24 primary schools and 25 high schools	10,657	7–17	53.4	46.6	The Children's Depression Inventory (CDI) DSM‐IV oriented	Depression (CDI cut‐off scores of ≥19) = 23.3%	Depression (CDI cut‐off scores of ≥19) = 24.7%	Depression (CDI cut‐off scores of ≥19) = 23.9%
28.	Wichaidit et al. (45)	Thailand	CS Cross‐sectional survey using multi‐stage cluster sampling of students in public and private schools in urban and rural areas, conducted in 2016 as part of the National School Survey	38,186	12–17	45.4	55.9	Patient Health Questionnaire‐2 (PHQ‐2)—modified for this study (students who answered “Yes” to the depressed mood and/or suicidal thoughts questions on the PHQ‐2, detected with major depression and/or suicidal ideation according to DSM‐IV criteria, respectively)	Depressed mood = 12.3% Suicidal ideation = 8.4%	Depressed mood = 13.9% Suicidal ideation = 9.5%	Years 7 and 9 Depressed mood: 11.6% Suicidal ideation: 8.9% Year 11 Depressed mood: 15.2% Suicidal ideation: 9.4%
29.	Zhong et al. (46)	China	CS Community Study using multistage, stratified cluster sampling of children and adolescents from randomly selected communities and households	3582	6–14	52.4	47.6	The Mini International Neuropsychiatric Interview for Children and Adolescents (MINI‐KID) DSM‐IV oriented	Any depressive disorder = 2.0% Major depressive disorder = 1.1% Dysthymia = 0.3% Minor depressive disorder = 0.6%	Any depressive disorder = 3.6% Major depressive disorder = 1.8% Dysthymia = 0.5% Minor depressive disorder = 1.3%	Any depressive disorder = 2.8% Major depressive disorder = 1.4% Dysthymia = 0.4% Minor depressive disorder = 0.9%
Both anxiety and depressive symptoms (22 studies)											
30.	Abubakar‐Abdullateef et al. (47)	Nigeria (Northwest)	CS Multistage sampling of children from 3 public schools	200	5–19	100.0	‐	The Schedule for Affective Disorders and Schizophrenia for School‐aged Children Present and Lifetime Version (K‐SADS‐PL) DSM‐IV oriented	‐	‐	Any psychiatric condition: 37.0% Depression: 8.1% Mania: 1.0% Separation anxiety: 14.0% Generalized anxiety disorder: 8.1% Obsessive compulsive disorder: 2.5% Posttraumatic stress disorder: 4.0% Agoraphobia: 3.0% Social phobia: 2.5%
31.	Al‐Jawadi and Abdul‐Rhman (48)	Iraq	CS Systematic random sampling of children and families from 2 primary community health care centers in Mosul, Iraq	3079	1–15	55.1	44.9	Standardized questionnaire including diagnostic criteria taken from DSM‐IV‐TR	Separation anxiety disorder = 4.2% Specific Phobia = 2.1% Post‐Traumatic Stress disorder = 7.8% Depression = 1.2%	Separation anxiety disorder = 4.5% Specific Phobia = 4.9% Post‐Traumatic Stress disorder = 13.8% Depression = 1.9%	Separation anxiety disorder = 4.3% Specific Phobia = 3.3% Post‐Traumatic Stress disorder = 10.5% Depression = 1.5%
32.	Anselmi et al. (49)	Brazil	CS 1993 Brazilian Birth Cohort Study‐cohort follow‐up during 2004–2005; home visits conducted	479	11–12	49.7	50.3	The Development and Well‐Being Assessment for Children and Adolescents parental version (DAWBA) DSM‐IV and ICD‐10 oriented	‐	‐	At least one psychiatric disorder according to DSM‐IV or ICD‐10: 10.8% Any anxiety disorder: 6.0% (DSM‐IV); 6.2% (ICD‐10) Separation anxiety disorder: 0.7% (DSM‐IV); 0.8% (ICD‐10) Specific phobia: 1.4% (DSM‐IV and ICD‐10) Social phobia: 0.1% (DSM‐IV and ICD‐10) Obsessive compulsive disorder: 0.1% (DSM‐IV and ICD‐10) Generalized anxiety disorder: 1.4% (DSM‐IV and ICD‐10) Posttraumatic stress disorder: 0.1% (DSM‐IV) Other anxiety disorder: 2.2% (DSM‐IV); 2.3% (ICD‐10) Any depressive disorder: 1.6% (DSM‐IV and ICD‐10) Major depression: 1.6% (DSM‐IV); 0.9% (ICD‐10) Other depressive disorder: 0.1% (DSM‐IV); 0.7% (ICD‐10) Agoraphobia: 0.1% (ICD‐10)
33.	Benjet et al. (50)	Mexico	CS National Mental Health Survey of adolescents selected from a stratified multistage area probability sample	3005	12–17	47.9	52.1	The World Mental Health version of the Adolescent Composite International Diagnostic Interview (WMH‐CIDI‐A) DSM‐IV (12‐month prevalence) oriented	Any psychiatric disorder (DSM‐IV) prevalent over 12‐month: 1.0%	Any psychiatric disorder (DSM‐IV) prevalent over 12‐month: 1.38%	Any anxiety disorder: 29.8% Panic disorder: 1.6% Generalized anxiety disorder: 0.5% Agoraphobia: 3.6% Social phobia: 11.2% Specific phobia: 20.9% Separation anxiety: 2.6% Posttraumatic stress disorder: 1.0% Any mood disorder: 7.2% Major depressive disorder: 4.8% Dysthymia: 0.5% Bipolar disorder: 2.5%
34.	Cortina et al. (51)	South Africa	CS Stratified random sampling of primary students from 10 primary schools	1025	10–12	50.8	49.2	The Youth Self Report (YSR) anxious/depressed scale DSM‐IV oriented	‐	‐	YSR scores in the clinical range of anxious/depressed scale: 14.1%
35.	dos Santos et al. (52)	Brazil	CS Part of cohort study of children randomly selected through stratified sampling within city of Salvador in 2001	349	4–6	54.7	45.3	The Child Behavior Checklist (CBCL) DSM‐IV oriented			Internalizing problems (based on clinical scores of CBCL) = 9.7% Anxiety/depression (based on clinical scores of CBCL) = 8.3%
36.	Hiscox et al. (53)	South Africa	CS Community sample of adolescents in Khayelitsha area of Cape Town who were recruited from four randomly selected high schools	797	13–17	37.8	62.2	Child PTSD Symptom Scale—Self Report for DSM‐5 (CPSS‐SR‐5) Center for Epidemiological Studies Depression Scale for Children (CES‐D‐10) (cut‐off scores for the CES‐D‐10 were not explicitly mentioned in the study)	Posttraumatic stress disorder: 20.4%	Posttraumatic stress disorder: 32.0%	Posttraumatic stress disorder: 27.6% Depressive symptoms: Not explicitly stated as percentages, but higher mean scores in females than males (males: 7.34, females: 8.19)
37.	Jiang et al. (54)	China (rural areas: Shaanxi, Gansu Shanghai and Suzhou)	Large‐scale epidemiological study in a sample of children and adolescents in Rural China, using multi‐stage sampling, national and regional surveys	53,421	9–16	52.0	48.0	Mental Health Test (MHT), which is derived from the Children's Manifest Anxiety Scale (CMAS) CFPS survey measured depression using the Center for Epidemiologic Studies Depression Scale (CES‐D)	Generalized anxiety: 4% Depression: 19%	Generalized anxiety: 6% Depression: 22%	Generalized anxiety: 6% Depression: 20%
38.	Kariuki et al. (55)	Kenya (Mombasa)	CS Community sample of families with children aged 3–5, randomly selected through the Kilifi Health and Demographic Surveillance System (KHDSS) across Kenyan rural area	3273	3–5	51.0	49.0	Child Behavior Checklist (CBCL) DSM‐IV oriented	Internalizing problems (based on CBCL): 23%	Internalizing problems (based on CBCL): 22%	Internalizing problems (based on CBCL): 22% Anxious/depressed (based on CBCL): 12.7%
39.	Khaleghi et al. (56)	Iran (Tehran)	CS Multistage (cluster and stratified) random sampling of children and adolescents from 350 clusters from all district of Tehran; 6 households randomly selected from each cluster	2095	6–18	51.4	48.6	The Schedule for Affective Disorders and Schizophrenia for School‐Age Children/Present and Lifetime Version (K‐SADS‐PL) DSM‐IV (semi‐structured Interview) oriented	Any psychiatric disorders: 30.5% (95% CI)	Any psychiatric disorders: 25.7% (95% CI)	Any psychiatric disorders: 28.2% (95% CI) Any mood disorders: 2.8% Depressive disorders: 2.3% Mania: 0.4% Hypomania: 0.7% Any anxiety disorders: 21.9% Panic disorder: 0.2% Separation Anxiety Disorder: 6.9% Social Phobia: 1.9% Specific Phobia: 9.9% Agoraphobia: 5.2% Generalized anxiety disorder: 2.8% Obsessive compulsive disorder: 7.8% Post‐Traumatic Stress disorder: 0.4%
40.	Kinyanda et al. (57)	Uganda (north‐east)	CS Multistage sampling of children and adolescents from a random selection of 210 households from each county across 4 districts of northeast Uganda	897	10–19	47.1	52.9	The MINI International Neuropsychiatric Interview for children and adolescents (MINI‐KID) DSM‐IV oriented	‐	‐	DSM‐IV mental disorder syndromes: Depressive disorder syndromes: 11.6% Anxiety disorder syndromes: 27.6%
41.	Maalouf et al. (58)	Lebanon	CS Community multistage cluster sampling of households from different areas within Beirut	510	11–17	56.1	43.9	The Development and well‐being assessment (DAWBA) DSM‐IV and ICD‐10 oriented	Emotional disorders = 13.6% Mood disorders = 4.5% Anxiety disorders = 10.8%	Emotional disorders = 21.4% Mood disorders = 9.4% Anxiety disorders = 16.1%	Emotional disorders = 17.1% Mood disorders = 6.7% Anxiety disorders = 13.1%
42.	Macul Ferreira de Barros et al. (59)	Brazil	Cohort study data from the Brazilian High‐Risk Study for Psychiatric Disorders, which included children from 57 public schools, collected at baseline and 3‐year follow‐up	1877	10–13 years at baseline 13–16 years at follow‐up	56.2	43.8	Developmental and Well‐Being Assessment (DAWBA) (cut‐off scores for the DAWBA were not explicitly mentioned in the study)			Baseline: Anxiety disorder = 12.0% Affective disorder: 3.6% Follow‐up (3 years later): Follow‐up prevalence were not explicitly stated in the text
43.	Nackers et al. (60)	Kenya Cambodia Uganda	CS Community sample of children aged 6 to 36 months in low‐resource settings	215 148 142	6 to 36 months	57.2 57.4 54.2	42.80 42.6 45.8	Psychological Screening for Young Children aged 6 to 36 months (PSYCa 6–36) (PSYCa 6–36 cut‐off scores to differentiate children with CGIS score of >1 vs. 1)			Psychological difficulties = 5.1% = 8.7% = 10.5%
44.	Sabet et al. (61)	South Africa	CS Cohort study Data collection drawn from the Birth to Twenty (BT20) cohort study when children were aged 11 to 12 years	1029	11–12	45.4	54.6	The Youth Self‐Report (YSR) DSM‐IV oriented	‐	‐	YSR internalizing problems = 41.2% YSR anxious/depressed scale = 23.2% YSR withdrawn/depressed scale = 1.4%
45.	Shen et al. (62)	China	CS Two‐stage, stratified sampling of children and adolescents from 13 randomly selected urban and rural schools in Hunan, China	17,071	6–16	51.8	48.2	The Mini International Neuropsychiatric Interview for Children and Adolescents (MINI‐KID) DSM‐IV oriented	Any psychiatric disorders = 11.79% Generalized anxiety disorder = 1.29% Separation anxiety disorder = 0 Post‐Traumatic Stress disorder = 0.01% Obsessive compulsive disorder = 0.51% Social Phobia = 0.07% Specific Phobia = 0.67% Major depressive disorder = 0.34% Dysthymia = 0.1% Mania = 0.25%	Any psychiatric disorders = 7.55% Generalized anxiety disorder = 2.28% Separation anxiety disorder = 0.04% Post‐Traumatic Stress disorder = 0.04% Obsessive compulsive disorder = 0.64% Social Phobia = 0.07% Specific Phobia = 0.81% Major depressive disorder = 0.90% Dysthymia = 0.24% Mania = 0.04%	Any psychiatric disorders = 9.74% Generalized anxiety disorder = 1.77% Separation anxiety disorder = 0.02% Post‐Traumatic Stress disorder = 0.02% Obsessive compulsive disorder = 0.57% Social Phobia = 0.07% Specific Phobia = 0.74% Major depressive disorder = 0.61% Dysthymia = 0.17% Mania = 0.15%
46.	Panter‐Brick et al. (63)	Afghanistan	CS Stratified random sampling of students attending 25 government‐operated schools in 3 regions	1011	11–16	49.8	50.2	The Birleson Depression Self‐Rating Scale (DSRS)—cutoff ≥ 15 (depressed) Children's Revised Impact of Events Scale (CRIES‐13)—cutoff ≥ 17 (high range) DSM‐IV and ICD‐10 oriented	Anxiety (based on CRIES cutoff scores ≥17) = 21.1%	Anxiety (based on CRIES cutoff scores ≥17) = 26.8%	Depression (based on DSRS cutoff scores ≥15) = 22.2% Anxiety (based on CRIES cutoff scores ≥17) = 30.9%
47.	Petresco et al. (64)	Brazil (Pelotas)	CS Cohort sample of all hospital births in city of Pelotas between January 1‐December 31, 2004; survey carried out at 5th follow‐up with children of the Pelotas birth Cohort at age 6	3585	Age 6 only	51.3	48.7	The Development and Well‐Being Assessment (DAWBA) DSM‐IV and ICD‐10 oriented	At least one psychiatric disorder according to DSM‐IV or ICD‐10 = 14.4% Any anxiety disorder: 8.8% (DSM‐IV); 8.4% (ICD‐10) Any depressive disorder: 1.5% (DSM‐IV and ICD‐10)	At least one psychiatric disorder according to DSM‐IV or ICD‐10 = 11.5% Any anxiety disorder: 8.8% (DSM‐IV and ICD‐10) Any depressive disorder: 1.1% (DSM‐IV and ICD‐10)	At least one psychiatric disorder according to DSM‐IV or ICD‐10 = 13% Any anxiety disorder: 8.8% (DSM‐IV); 8.6% (ICD‐10) Separation anxiety disorder: 3.2% (DSM‐IV); 2.9% (ICD‐10) Specific phobia: 5.4% (DSM‐IV and ICD‐10) Social phobia: 0.1% (DSM‐IV and ICD‐10) Obsessive compulsive disorder: 0.2% (DSM‐IV and ICD‐10) Generalized anxiety disorder: 0.2% (DSM‐IV and ICD‐10) Posttraumatic stress disorder: 0.8% (DSM‐IV); 0.7% (ICD‐10) Other anxiety disorder: 0.1% (DSM‐IV and ICD‐10) Agoraphobia: 0.03% (DSM‐IV and ICD‐10) Any depressive disorder: 1.3% (DSM‐IV and ICD‐10) Minor depression: 1.2% (DSM‐IV); 0.5% (ICD‐10) Major depression: 0.08% (DSM‐IV and ICD‐10)
48.	Pillai et al. (65)	India (Goa)	CS Population‐based survey of all eligible adolescents from 10 randomly selected rural and urban communities	2048	12–16	50.3	49.7	The Development and Well‐Being Assessment (DAWBA) DSM‐IV and ICD‐10 oriented			At least one psychiatric disorder according to DSM‐IV (95% CI) = 1.81% Any anxiety disorders = 1.0% Any depressive disorders = 0.5%
49.	Weiss et al. (66)	Vietnam	CS National survey using cluster sampling of children and adolescents from urban, near‐urban, and rural communities in 10 provinces	591	6–16	50.0	50.0	Child Behavior Checklist (CBCL Or Youth Self‐Report (YSR) DSM‐IV oriented			CBCL/YSR internalizing problems = 18.5%–20.0% CBCL/YSR anxious/depressed scale = 7.3%–8.6% CBCL/YSR withdrawn/depressed scale = 5.1%–6.3%
50.	Xu et al. (67)	China	CS Random sampling of students from 3 junior and 3 high schools in Hefei, eastern China	3019	13–18	51.7	48.3	Beck Depression Inventory (BDI) Self‐rating Anxiety Scale (SAS) Both DSM‐IV oriented			Depression symptoms (BDI cut‐off scores of ≥14) = 11% Anxiety symptoms (SAS cut‐off scores of ≥50) = 21.5%
51.	Idris et al. (68)	Malaysia	CS Stratified multistage cluster sampling of students from primary schools Year 1/2; and secondary schools in Form 1 and 2	154	7–14	52	48	Strengths and Difficulties Scores (SDQ): Parent‐, Teacher‐ and Child‐reported versions Children will be classified as abnormal when SDQ total difficulties scores were between 17 and 40			Total difficulties including emotional problems (parent‐reported) = 8.5% Total difficulties including emotional problems (teacher‐reported) = 9.3% Total difficulties including emotional problems (child‐reported) = 3.9% Emotional problems (parent‐reported) = 8.3% Emotional problems (teacher‐reported) = 3.9% Emotional problems (child‐reported) = 3.9%

### Study Settings

The 51 studies represented 23 out of the 138 countries categorized as having low‐ and middle‐income economies by the World Bank in 2018. Specifically, three studies covered populations classified as low‐income (Afghanistan, Malawi, Uganda, and Tanzania), eight were classified as lower‐middle‐income (Cambodia, Egypt, India, Kenya, Pakistan, Nigeria, Sri Lanka, Sudan, and Vietnam) and nine were classified as upper‐middle‐income (Brazil, China, Iran, Iraq, Lebanon, Malaysia, Mexico, South Africa, Thailand, and Turkey).

Twenty‐four studies recruited participants through multistage sampling of primary and secondary schools. In 14 studies, participants were recruited from a population‐based setting in which they were selected through multistage random selection of households. One study recruited children through four healthcare centers in the city. The healthcare centers were national centers for vaccination initiated by the national government, whereby parents were required to bring their children for vaccination and were invited to participate in a survey alongside this. Two studies assessed birth cohort participants through home visit follow‐ups.

One specific cohort study conducted a follow‐up with participants who were invited to be assessed at collection sites that were one of two hospitals in two different cities of the country. In another cohort study, participants were invited for assessment at a research clinic, and if not feasible, assessments were conducted through home visits. The experiences of the children and adolescents in the above studies still represented those in the general community and therefore the studies were included in the review.

### Study Designs

All but five of the studies involved cross‐sectional surveys. These five studies used a cohort design and reported sample data using cross‐sectional methods where two or more distinct age cohorts were tracked and assessed over a number of years.

### Participants

Studies included a total of 208,842 participants aged between 1 and 20 years (49% female and 51% male) who were assessed for anxiety and depressive symptoms. One study had only one participant aged 20 years old and attending the same high school as the rest of the sample. Hence, it was considered appropriate to include this study in the review. Three studies assessed females only, whereas one study assessed males only. In these four single‐gender studies, the recruitment of participants was carried out using multistage random sampling. Due to the large sizes of populations being tested, the selection of participants underwent two stages: firstly, the selection of eligible clusters, then the selection of samples of individuals from these clusters. This sampling step in the four gender‐specific studies was done in a bid to ensure that the sample fell within the age range for inclusion in the study, hence it was deemed justifiable to include these studies in the review. Representing 48% of the total sample, participants aged 1 to 19 years were examined for both anxiety and depression related problems. Only 5% of participants, ranging from the age of 3 to 19 years, were examined for anxiety‐related problems alone, whereas 50% of participants aged 3 to 20 years were assessed for depression‐related problems only.

### Methods Used to Assess Emotional Problems

Across the 25 studies that assessed only depression‐related problems, eight different standardized instruments were used with a total sample of 104,163 participants. In the four studies assessing only anxiety symptoms, three different standardized measures were used among the sample of children and adolescents (*N* = 4659). The 22 studies which assessed both anxiety and depression‐related problems used 15 different standardized instruments that measured emotional problems (including anxiety and depression) among a sample of 100,020 children and adolescents.

#### Assessments of Depression‐Related Problems

The most widely used instrument was the Children's Depression Inventory (CDI), which was utilized across seven studies with cut‐off scores ranging from 12 to 24 for the screening of depression. The second most used instrument was the Center for Epidemiological Studies Depression Scale (CES‐D), which was used across four studies with cut‐off scores ranging from 16 to 22, depicting elevated depressive symptoms. The Kiddie Schedule for Affective Disorders and Schizophrenia for School Age Children‐Present and Lifetime Version (K‐SADS‐PL) and the Epidemiological Version 5 (K‐SADS‐E), semi‐structured interviews based on DSM‐III‐TR and DSM‐IV, were used in three studies. Additionally, based on DSM‐IV criteria, the MINI International Neuropsychiatric Interview for Children and Adolescents (MINI‐KID) and a structured clinical diagnostic interview were used in four studies. The Beck Depression Inventory (BDI) was used in three studies with cut‐off scores ranging from 8 to 19. The Patient Health Questionnaire (PHQ‐9) for depression was used in three studies: two studies used cut‐off scores ≥5 to depict depression, while the other study used a diagnostic algorithm of the PHQ‐9 to screen for depression (i.e., the presence of two or more depressive symptoms, with at least one symptom being depressed mood). Additionally, one study used the modified version of the Patient Health Questionnaire‐2 (PHQ‐2): the depression screening question in that study was based on one of the two items that constituted the PHQ‐2 screening tool. Another two studies used the Structured Clinical Interview for DSM‐IV Disorders (SCID) for the diagnosis of depression.

Two studies used a two‐stage design. In Adewuya et al. (23), children (*n* = 1095) were firstly screened using the BDI and based on their scores, a subset (*n* = 454) were further interviewed for DSM‐IV diagnosis of depression with the K‐SADS‐E. In Demir et al. (25), participants (*N* = 1482) were screened using the CDI and based on the results, a subset (*n* = 320) was interviewed with the K‐SADS‐PL. El‐Missiry et al. (26) used both the CDI and the Non‐patient version of the Structured Clinical Interview for DSM‐IV axis‐I disorders (SCID‐I/NP) for the diagnosis of depression.

#### Assessments of Anxiety‐Related Problems

Two studies used the MINI‐KID, a brief structured interview for the major Axis I psychiatric disorders in DSM‐IV and DSM‐IV‐TR criteria with specific algorithms for diagnosis of depression. One study used the Screen for Child Anxiety Related Emotional Disorders (SCARED) with cut‐off scores ≥21 depicting depressive symptoms. Another study (20) used a 2‐stage design. Participants (*n* = 1482) were screened in stage 1 with the Social Anxiety Scale for Children‐Revised (SASC‐R) and the Capa Social Phobia Scale for Children and Adolescents (CSPSCA). Based on the results of stage 1, stage 2 involved diagnostic interviews of the sample (*n* = 324) using the K‐SADS‐PL.

#### Assessments of Emotional Problems (Anxiety and Depressive Symptoms)

The most widely used instrument across five studies was the Development and Well‐Being Assessment for Children and Adolescents (DAWBA). Based on DSM‐IV and ICD‐10 criteria, the DAWBA is an integrated package of questionnaires, interviews and rating techniques designed to generate best‐estimate psychiatric diagnoses in children and adolescents. In these studies, information including open‐ended questions was gathered from both young people and their parents. This was then reviewed by mental health professionals to verify or overrule the generated diagnoses. Two studies used the Youth Self Report (YSR)—anxious/depressed scale, making use of cut‐off scores ≥60 to differentiate young people in the clinical range for anxiety and depression. Two studies made use of the Child Behaviour Checklist (CBCL), using cut‐off scores ≥60 to depict clinical internalizing problems among young people. One study used both the YSR and the CBCL to investigate internalizing problems of anxiety and depression among children and adolescents. Two studies used the MINI‐KID, two studies made use of the K‐SADS‐PL, and one study used a standardized diagnostic criteria questionnaire based on DSM‐IV‐TR. These structured diagnostic interviews were used to assess current and past psychopathology pertaining to anxiety and depressive problems.

One study used the Adolescent version of the World Mental Health Composite International Diagnostic Interview (WMH‐CIDI‐A), which is a structured diagnostic schedule for mental disorders including anxiety and depression. Two studies used two separate scales to assess depression and anxiety severity. One study used the Birleson Depression Self Rating Scale (DSRS) with cut‐off scores ≥15 and the Children's Revised Impact of Events Scale (CRIES‐13) with cut‐off scores ≥17 to assess depression and anxiety severity respectively. The other study used BDI with cut‐off scores ≥14 to assess depression and the self‐rating Anxiety Scale (SAS) with cut‐off scores ≥50 to classify as high anxiety. One study used the Child PTSD Symptom Scale—Self Report for DSM‐5 (CPSS‐SR‐5) for assessing posttraumatic stress symptoms, and CES‐D‐10 for assessing depressive symptoms in adolescents. One study used the Mental Health Test (MHT), which is derived from the Children's Manifest Anxiety Scale (CMAS) for assessing the anxiety symptoms, and CES‐D‐10 for assessing depressive symptoms in children and adolescents.

### Prevalence Estimates of Emotional Problems

The prevalence of emotional problems from the 51 studies between the ages of 1 to 20 years were extracted. In some studies, authors reported prevalence rates for the individual depression and anxiety disorders in addition to “any anxiety diagnosis,” or “any depressive diagnosis.” In these cases, the “any anxiety diagnosis” and “any depressive diagnosis” figures were used for the total prevalence. The general and specific individual prevalence rates of the diagnoses and/or severity of emotional problems are presented in full in online supplement.

#### Prevalence of Depression‐Related Problems

Twenty‐five studies, including 104,163 child and adolescent participants, provided data based on standardized instruments and/or diagnostic interviews on depression‐related problems only. Eighteen studies reported data from standardized measures depicting mild to severe depression and five studies reported data from diagnostic interviews depicting any depressive disorder, including major depressive disorder, dysthymia, and depressive disorders not otherwise specified. Two studies reported data from both standardized measures and diagnostic interviews during the different stages of the related studies. Depression had a prevalence estimate ranging from 1% to 58% among children and adolescents in LMICs.

#### Prevalence of Anxiety‐Related Problems

Four studies, including 4659 child and adolescent participants, provided data based on standardized instruments and/or diagnostic interviews on anxiety‐related problems only. One study reported data from standardized measures depicting mild to severe anxiety, and two studies reported data from diagnostic interviews depicting any anxiety disorder, including generalized anxiety disorder, separation anxiety disorder, panic disorder, social phobia (social anxiety disorder), panic with agoraphobia, specific phobia, obsessive compulsive disorder, and PTSD. One study reported data from both standardized measures and diagnostic interviews during the different stages of the related study. The prevalence estimates for anxiety‐related problems ranged from 1% to 30% among children and adolescents in LMICs. Types of anxiety problems with the highest prevalence estimates related to symptoms of generalized anxiety (18.2%), separation anxiety (14.0%), posttraumatic stress (32.0%), specific phobia (20.9%), and social phobia/social anxiety (20.2%).

#### Prevalence of Emotional Problems (Both Anxiety and Depression)

Twenty‐two studies, including 100,020 child and adolescent participants, provided data based on standardized instruments and/or diagnostic interviews on emotional problems, including both anxiety‐ and depression‐related problems. Twelve studies reported data from standardized measures depicting mild to severe emotional problems, and five studies reported data from diagnostic interviews depicting any anxiety and depression disorders based on DSM or ICD diagnostic criteria. Five studies reported data from an integrated package of standardized questionnaires and diagnostic interviews. The prevalence estimates for emotional problems ranged from 1% to 41% among children and adolescents in LMICs.

## DISCUSSION

This review examined 51 studies reporting on emotional problems experienced by 208,842 children and adolescents living in 23 LMICs. Investigating the prevalence of emotional problems is considered to be the first step towards determining the magnitude of a problem. Emotional problems of anxiety and depression in LMICs are investigated through a wide range of methodological approaches. Hence, comparability between the methodology of these studies is limited owing to the different standardized instruments and diagnostic interviews used for assessment. The measures used in LMICs seem comparable to those used in HICs. Some of the most widely used standardized instruments in LMICs include the CDI, BDI, and CES‐D. While the K‐SADS‐PL, K‐SADS‐E, MINI‐KID, and the DAWBA, were the most used diagnostic interviews across the studies. In total, studies measuring anxiety used three different instruments, whereas studies measuring depression used eight different assessments, and studies measuring both anxiety and depression used 15 different measures. All of the methodological approaches in the studies were derived from the two most used diagnostic systems: the DSM and the ICD.

A few studies used a one‐stage design in which diagnostic interviews were used to assess an entire sample of the population. This design provides a strong methodological basis for assessing prevalence rates. However, it is costly and time‐consuming, limiting its feasibility. To address these limitations, some studies used a two‐stage design whereby the entire sample is screened for emotional problems and those with high/low scores are further selected for the diagnostic phase. With reliable screening methods and appropriate cut‐offs, and clinician‐led or trained interviewers, prevalence rates may be estimated effectively. However, these important elements are found to be frequently inconsistent across studies and thus may impact on the resulting prevalence rates (69). Recent research underscores the need for standardized screening protocols and consistent methodologies to improve the accuracy of prevalence estimates in diverse settings (14, 70).

In this systematic review, an estimated prevalence ranging from 1% to 58% for depression, and from 1% to 30% for anxiety was found. An overall prevalence for emotional problems ranged from 1% to 41%. These findings for anxiety are comparable to the reported prevalence estimates of up to 31.9% for anxiety disorders in the United States (3). However, the review's findings for depression and emotional problems are much higher than Merikangas et al.’s (3) reported prevalence rate of up to 11.7%. Since most children and adolescents across the world live in LMICs, these prevalence rates translate into an enormous number of affected young people, and the impact may be even more detrimental (1, 71, 72). Emotional problems limit an individual's psychosocial functioning, diminishing their quality of life, and are frequently associated with substantial burdens and psychological, medical and financial costs not only for the individuals and their families, but also for society as a whole (73). Recent trends indicate that mental health conditions among adolescents constitute a significant portion of the global disease burden, emphasizing the need for targeted interventions to address these challenges (74, 75). Additionally, recent research further highlights the compounded effects of the COVID‐19 pandemic on the mental health of young people in LMICs, exacerbating existing challenges and increasing the prevalence of anxiety and depression (76).

It is important to note that 15 studies included in the review covered Sub‐Saharan African countries, where rates of emotional problems are particularly elevated (6, 77, 78, 79). Studies have further documented the high prevalence of these issues in Africa, with some findings indicating that anxiety disorders affected up to 47% of the population during the COVID‐19 pandemic (80), and depression rates have been found to range from 26.9% to 45.2% among adolescents in various regions of Uganda (81). Moreover, all of the studies included in the review form part of the Global South, a term usually denoted for countries with low‐ and middle‐income and which are often politically and/or culturally marginalized (82). An increased prevalence of emotional problems in the Global South may relate to social, cultural, economic and environmental risk factors, which can impact on children and adolescents directly or indirectly (83, 84).

The prevalence of emotional problems in LMICs seems to be higher than in HICs. While it was beyond the scope of the present study to examine factors that put young people at risk of developing anxiety and depression, studies have suggested that existing and emerging social, cultural and economic difficulties in LMICs increase the vulnerability to these different types of anxiety and depressive symptoms (85, 86, 87). It could be speculated that the higher prevalence relates to the challenges in mental health policy faced by LMICs (13). Poorer social and environmental circumstances, poor public awareness, low political willingness, mental health stigma, shortage of mental health resources and biased cultural values towards children and adolescents' emotional wellbeing may contribute to a heightened risk of emotional problems. Knowledge of the types of emotional problems and their impact on the lives of children and adolescents were evidenced in the review's studies. One key risk factor to this increased vulnerability may relate to the higher number of left‐behind children and adolescents in LMICs by one or both migrant parents, with 85% of these young people experiencing an increased risk of anxiety and 52% having an increased risk of depression (88). Recent research continues to underscore the negative impact of parental migration, revealing significant associations between parental absence and increased emotional and behavioral problems in left‐behind children (89, 90). Experience of early parental separation and insecure attachment also increase emotional problems, such as low self‐esteem, a lack of self‐confidence, loneliness, depression, emotional instability, and social anxiety (91). Moreover, an increasing number of young people are being orphaned due to high rates of mortality from poor health conditions such as HIV/AIDS, tuberculosis, malaria and natural disasters across African and Asian countries (92).

Another related risk factor pertains to young people's experiences of war, conflict, and violence in LMICs. The risk of internalized difficulties has been found to be 7.1 times higher in children and adolescents experiencing war‐related violence (93). Since 2010, LMICs have witnessed a dramatic increase in fragility, conflict, and violence (94). The deleterious effects of living under armed conflict are profound for children and adolescents (95, 96). Children who are exposed either directly or indirectly to conflict suffer psychological harm that persists across their life course and beyond, and even to subsequent generations born after the conflict has ended (97). Prevalence rates of up to 87% have been found for symptoms of PTSD among youth exposed to traumatic experiences in LMICs (6), compared to rates up to 8% having been found in an HIC (98). Poverty is another important risk factor, as many of those who live below the poverty line are part of the populations of LMICs (99). Poverty is strongly associated with emotional problems (100). Recent studies have shown that the multidimensional nature of poverty, including factors such as poor access to education, inadequate housing, and food insecurity, significantly contributes to mental health issues among children and adolescents in LMICs (101). This relationship is multi‐layered, with overlapping factors such as malnutrition, poor physical health, higher mortality rates, and crime, which substantially increase the risk of mental illness (100, 102). This is compounded by the fact that poverty often reduces access to care and availability of treatment services, which in turn creates a perpetuating cycle of mental illness (103). Studies across Brazil, India and Zimbabwe have shown that rates of emotional problems are about twice as high in lower‐income than higher‐income groups, supporting the notion that poverty and other adverse social determinants are high risk factors for emotional problems such as anxiety and depression (104).

Child maltreatment in LMICs is also an important risk factor, with associated pathways involving poverty, caregiver mental health distress, HIV/AIDS and sociocultural variations in family structures and attitudes (105, 106). Such adverse childhood experiences (ACEs; a constellation of exposures including abuse, neglect, and household challenges) are linked to negative long‐term effects of childhood trauma with poorer health across the life course (107, 108). However, cultural and social structures including gender and age inequalities not only predispose individuals to being victims of abuse but also make it difficult for victims to respond safely to the abuse. Cultural norms across some LMICs dictate and restrict child behaviors, making it easier for the adults to take advantage of children and making it difficult for children to seek help (109). Recent studies have shown that interventions focusing on enhancing caregiver capacities and community awareness can mitigate some of the impacts of these risk factors (75, 110). Hence, further research is required for addressing the coverage of risk factors of emotional problems during the developmental periods of childhood and adolescence in LMICs. Additionally, many of the included studies did not disaggregate findings by specific age groups, such as younger children versus adolescents. This limited the ability to explore developmental differences in the prevalence of emotional problems. Future research would benefit from age‐specific reporting to better understand how emotional difficulties may vary across developmental stages.

This systematic review indicates significant variability in reported prevalence estimates of anxiety, depression, and comorbid emotional problems among children and adolescents across LMICs. It is unclear whether the heterogeneity of frequency of emotional problems between studies reflects true differences or the influences of other factors. Despite the fact that studies used reliable psychometric tools, there was variability in assessment such that some studies relied on clinical interviews based on DSM or ICD diagnostic criteria, whereas others used self‐ or parent‐reported rating scales. Moreover, the transferability of western assessment measures to non‐western societies should be treated with caution, as biases may still occur in relation to cross‐cultural understanding, interpretation and translation (111).

The review's studies included data from children from a broad age band. However, it is possible that the transition from childhood to adolescence within different cultural milieus might influence the prevalence of estimates of emotional problems. Emotional development and symptom presentation differ significantly between younger children and adolescents, which may contribute to inconsistent prevalence estimates across studies. Furthermore, cultural norms shape how emotional distress is expressed and interpreted, which may influence both the accuracy of assessment tools and participants' willingness to disclose symptoms. In many LMICs, mental health stigma remains a significant barrier to open discussion and recognition of emotional problems, which can lead to underreporting and misclassification in research studies. Studies focusing on clinical or multimorbid populations were excluded to maintain consistency and comparability in prevalence estimates across general populations. However, this exclusion may limit insight into the burden of emotional problems among children with complex needs, potentially underestimating prevalence in high‐risk subgroups.

To address these challenges, national policies in LMICs should prioritize investment in culturally adapted diagnostic tools, the training of non‐specialist mental health workers in accurate identification practices, and the implementation of standardized data collection systems. Such policy reforms would support more consistent diagnosis and reporting, helping reduce misdiagnosis and address broader methodological challenges in mental health research.

## CONCLUSIONS

This systematic review attempted to decrease the knowledge gap related to emotional problems faced by young people in LMICs. Results indicate that emotional problems in LMICs are notable among children and adolescents. Reported prevalence estimates for anxiety and depression varied wildly among LMICs but were either comparable or higher than the reported rates in HICs. An explanation for this is that the majority of children and adolescents in LMICs might have been exposed to several risk factors, such as parental migration, conflict and violence, and poverty. These results are essential to service, training, and research planning in LMICs, and should be used in a manner that will promote sound and sustainable anxiety and depression prevention programmes for young people in LMICs.

## Supporting information

Supplementary Materials S1
