# Supplementary material for: Prevalence of Anxiety and Depression Among Children and Adolescents in Low‐ and Middle‐Income Countries—A Systematic Review
Source: Psychiatr Res Clin Pract. 2025 Jul 31;7(4):218–43. doi: 10.1176/appi.prcp.20250026 (PMC12710537; doi:10.1176/appi.prcp.20250026)
Supplement: Supplementary file 1 — Supplementary Materials S1 [file RCP2-7-218-s001.docx]

### Supplementary Materials 1: Search Strategy in Medline (PUBMED) and PsycINFO (EBSCO)

##### CONCEPTS used for systematic review:

1: children/adolescents

2: emotional problems/anxiety/depression

3: developing countries/Low- and Middle-Income Countries (LMICs)

4: study design

| **CONCEPT 1** | OR | **MeSH:** adolescent, child, childhood, minors, teens, youth, young (people, person, population) |
| --- | --- | --- |
|  |  | **Key words:** (child* OR adolescen* OR teen* OR minor OR youth* OR (young adj (people OR person OR population*))) |
| **CONCEPT 2** | OR | **MeSH:** Emotional problems, emotional disorders, emotional distress, emotional symptoms, emotional difficulties, anxiety, depression, mental health, mental health disorders, mental health problems, psychological problems, psychological disorders, psychological distress, psychological symptoms, psychological difficulties, depressive disorders, anxiety disorders, affective disorders, mood disorders, panic disorders, dysthymia, bipolar, agoraphobia, internalizing, internalising |
|  |  | **Key words:** (emotion* adj (problem* OR disorder* OR distress OR symptom* OR difficult* OR health)) OR anxi* OR depressi* OR (mental adj (health OR disorder* OR problem*)) OR (psycholog* adj (problem* OR disorder* OR distress OR symptom* OR difficult*)) OR (depress* disorder*) OR (anxiety disorder*) OR (affect* disorder*) OR (mood disorder*) OR (panic disorder*) OR (bipolar disorder*) OR (internalising disorder*) OR (internalizing disorder*) |
| **CONCEPT 3** | OR | **MeSH:** developing countries or low income countries or middle income countries or low middle income countries or africa or caribbean region or central america or latin America or south America or asia central or asia southeastern or asia western or asia eastern or asia pacific or indian ocean islands or pacific islands or melanesia or micronesia or west indies |
|  |  | **Key words:** (resource-limit* OR resource-poor OR low-resource* OR limited-resource* OR resource-constrain* OR constrained-resource* OR under-resourced OR poor-resource* OR resource-scarce OR scarce-resource* OR low-income OR middle-income OR lower-middle-income OR upper-middle-income OR LIC OR LMIC OR LALMIC OR LMAUMIC) OR (developing OR underdeveloped OR under-developed OR emerging OR less-developed OR least-developed OR less-economically developed OR least-economically developed OR less-affluent OR least-affluent) adj (country OR countries OR nation OR nations OR region OR regions OR economy OR economies OR world* OR population*) OR (Afghanistan* OR Albania* OR Algeria* OR American Samoa* OR Angola* OR Argentina* OR Armenia* OR Azerbaijan* OR Bangladesh* OR Belarus* OR Belize* OR Benin* OR Bhutan* OR Bolivia* OR Bosnia And Herzegovina* OR Botswana* OR Brazil* OR Bulgaria* OR Burkina Faso* OR Burundi* OR Cabo Verde* OR Cambodia* OR Cameroon* OR Central African Republic* OR Chad* OR China* OR Colombia* OR Comoros* OR Congo* OR Congo Rep* OR Costa Rica* OR Cote D’ivoire* OR Cuba* OR Djibouti* OR Dominica* OR Dominican Republic* OR Ecuador* OR Egypt* OR El Salvador* OR Equatorial Guinea* OR Eritrea* OR Ethiopia* OR Fiji* OR Gabon* OR Gambia* OR Georgia* OR Ghana* OR Grenada* OR Guatemala* OR Guinea* OR Guinea-Bissau* OR Guyana* OR Haiti* OR Honduras* OR India* OR Indonesia* OR Iran* OR Iraq* OR Jamaica* OR Jordan* OR Kazakhstan* OR Kenya* OR Kiribati* OR Korea* OR Kosovo* OR Kyrgyz Republic* OR Lao Pdr* OR Lebanon* OR Lesotho* OR Liberia* OR Libya* OR Macedonia FYR* OR Madagascar* OR Malawi* OR Malaysia* OR Maldives* OR Mali* OR Marshall Islands* OR Mauritania* OR Mauritius* OR Mexico* OR Micronesia, Fed. Sts.* OR Moldova* OR Mongolia* OR Montenegro* OR Morocco* OR Mozambique* OR Myanmar* OR Namibia* OR Nepal* OR Nicaragua* OR Niger* OR Nigeria* OR Pakistan* OR Panama* OR Papua New Guinea* OR Paraguay* OR Peru* OR Philippines* OR Romania* OR Russian Federation* OR Rwanda* OR Samoa* OR Sao Tome And Principe* OR Senegal* OR Serbia* OR Sierra Leone* OR Solomon Islands* OR Somalia* OR South Africa* OR South Sudan* OR Sri Lanka* OR St. Lucia* OR St. Vincent And The Grenadines* OR Sudan* OR Suriname* OR Swaziland* OR Syrian Arab Republic* OR Tajikistan* OR Tanzania* OR Thailand* OR Timor-Leste* OR Togo* OR Tonga* OR Tunisia* OR Turkey* OR Turkmenistan* OR Tuvalu* OR Uganda* OR Ukraine* OR Uzbekistan* OR Vanuatu* OR Venezuela* OR Vietnam* OR West Bank And Gaza* OR Yemen Rep* OR Zambia* OR Zimbabwe* OR Africa* OR Asia* OR Caribbean OR Central America* OR Latin America* OR South America* OR Melanesia* OR Micronesia* OR Polynesia*) |
| **CONCEPT 4** | OR | **MeSH:** epidemiologic studies/ or case-control studies/ or exp cohort studies/ or controlled before-after studies/ or cross- sectional studies/ exp longitudinal studies/ or prospective studies/ or retrospective studies/ |
|  |  | **Key words:** (epidemiologic* adj (study OR studies)) OR (case-based OR case-control OR case-comparison OR case-referent OR case-compeer) OR ((cohort OR incidence OR concurrent) adj (study OR studies)) OR ((prevalence OR cross-sectional) adj (study OR studies)) OR (cross-sectional adj (survey* OR analysis OR analyses)) OR ((cross-sectional OR disease frequency) adj (survey OR surveys)) OR (cohort analysis OR cohort analyses) OR ((follow-up OR followup) adj (study OR studies)) OR ((retrospective OR prospective OR observational) adj (study OR studies)) OR (longitudinal adj (study OR studies OR survey OR surveys)) |
| **AND** | | **CONCEPT 1 AND CONCEPT 2 AND CONCEPT 3 AND**  **CONCEPT 4** |
| **Results limited to English language and publications as from 01/01/2000 to 30/04/2024** | | |
| **Search: Keywords & Medical Subject Headings (MeSH)** | | |

**Search strategy (keywords) used in PSYCINFO (EBSCO):**

| **#** | **Searches** | **Hits** |
| --- | --- | --- |
| 1. | child* |  |
| 2. | adolescen* |  |
| 3. | teen* |  |
| 4. | Youth |  |
| 5. | Minor |  |
| 6. | Minors |  |
| 7. | young N1 (people or person* or population*) |  |
| 8. | OR/ 1 – 7 | 691,170 |
| 9. | emotion* N1 (problem* or disorder* or distress or symptom* or difficult* or health) |  |
| 10. | anxi* |  |
| 11. | depress* |  |
| 12. | mental N1 (health or disorder* or problem*) |  |
| 13. | psycholog* N1 (problem* or disorder* or distress or symptom* or difficult*) |  |
| 14. | depress* disorder* |  |
| 15. | anxiety disorder* |  |
| 16. | affect* disorder* |  |
| 17. | mood disorder* |  |
| 18. | panic disorder* |  |
| 19. | bipolar disorder* |  |
| 20. | internali#ing disorder* |  |
| 21. | OR/ 9 – 20 | 459,264 |
| 22. | AB (“developing country” or “developing countries” or “developing nation” or “developing nations” or less* W1 “developed country” or less* W1 “developed countries” or less* W1 “developed nation” or less* W1 “developed nations” or “third world” or “under developed” or “middle income” or “low income” or “underserved country” or “underserved countries” or “underserved nation” or “underserved nations” or “under served country” or “under served countries” or “under served nation” or “under served nations” or “underserved population” or “underserved populations” or “under served population” or “under served populations” or “deprived country” or “deprived countries” or “deprived nation” or “deprived nations” or poor* W1 country or poor* W1 countries or poor* W1 nation* or poor* W1 population*) |  |
| 23. | AB (resource-limit* or resource-poor or low-resource* or limited-resource* or resource-constrain* or constrain*- resource* or under-resource* or poor*-resource* or resource-scarce* or scarce*-resource* or low-income or middle-income or lower-middle-income or upper-middle- income or LIC or LMIC or LALMIC; LMAUMIC) N2 countr* or (developing or underdeveloped or under- developed or emerging or less-developed or least-developed or less-economically developed or least-economically developed or less-affluent or least-affluent) N2 (countr* or nation* or nations or region* or econom* or world or population*) |  |
| 24. | Afghanistan* or Albania* or Algeria* or American Samoa* or Angola* or Argentina* or Armenia* or Azerbaijan* or Bangladesh* or Belarus* or Belize* or Benin* or Bhutan* or Bolivia* or Bosnia And Herzegovina* or Botswana* or Brazil* or Bulgaria* or Burkina Faso* or Burundi* or Cabo Verde* or Cambodia* or Cameroon* or Central African Republic* or Chad* or China* or Colombia* or Comoros* or Congo* or Congo Rep* or Costa Rica* or Cote D'ivoire* or Cuba* or Djibouti* or Dominica* or Dominican Republic* or Ecuador* or Egypt* or El Salvador* or Equatorial Guinea* or Eritrea* or Ethiopia* or Fiji* or Gabon* or Gambia* or Georgia* or Ghana* or Grenada* or Guatemala* or Guinea* or Guinea-Bissau* or Guyana* or Haiti* or Honduras* or India* or Indonesia* or Iran* or Iraq* or Jamaica* or Jordan* or Kazakhstan* or Kenya* or Kiribati* or Korea* or Kosovo* or Kyrgyz Republic* or Lao Pdr* or Lebanon* or Lesotho* or Liberia* or Libya* or Macedonia, Fyr* or Madagascar* or Malawi* or Malaysia* or Maldives* or Mali* or Marshall Islands* or Mauritania* or Mauritius* or Mexico* or Micronesia, Fed. Sts.* or Moldova* or Mongolia* or Montenegro* or Morocco* or Mozambique* or Myanmar* or Namibia* or Nepal* or Nicaragua* or Niger* or Nigeria* or Pakistan* or Panama* or Papua New Guinea* or Paraguay* or Peru* or Philippines* or Romania* or Russian Federation* or Rwanda* or Samoa* or Sao Tome And Principe* or Senegal* or Serbia* or Sierra Leone* or Solomon Islands* or Somalia* or South Africa* or South Sudan* or Sri Lanka* or St. Lucia* or St. Vincent And The Grenadines* or Sudan* or Suriname* or Swaziland* or Syrian Arab Republic* or Tajikistan* or Tanzania* or Thailand* or Timor-Leste* or Togo* or Tonga* or Tunisia* or Turkey* or Turkmenistan* or Tuvalu* or Uganda* or Ukraine* or Uzbekistan* or Vanuatu* or Venezuela* or Vietnam* or West Bank And Gaza* or Yemen Rep* or Zambia* or Zimbabwe* |  |
| 25. | africa* or asia* or caribbean or central america* or latin america* or south america* or melanesia* or micronesia* or polynesia* |  |
| 26. | OR/ 22 – 25 | 554,718 |
| 27. | epidemiolog* |  |
| 28. | prevalence N1 (stud* or survey* or analys*) |  |
| 29. | epidemiologic* N1 (stud* or case-base or case-control or  case-comparison or case-refer?en*) |  |
| 30. | (cohort or incidence or concurrent) N1 stud* |  |
| 31. | cross-sectional N1 (stud* or survey* or analys*) |  |
| 32. | disease frequenc* N1 (survey* or stud*) |  |
| 33. | cohort analys* |  |
| 34. | (followup or follow-up) N1 stud* |  |
| 35. | (retrospective or prospective or observational) N1 stud* |  |
| 36. | longitudinal N1 (stud* or survey*) |  |
| 37. | OR/ 27 – 36 | 73,969 |
| 38. | **8 AND 21 AND 26** | 2160 |

**Search strategy used in MEDLINE (Pubmed)**

**MeSH terms search:**

| 1. | child | "child"[MeSH Terms] OR "child"[All Fields] |
| --- | --- | --- |
| 2. | adolescents | "adolescent"[MeSH Terms] OR "adolescent"[All Fields] OR "adolescents"[All Fields] |
| 3. | childhood | "Childhood"[Journal] OR "childhood"[All Fields] |
| 4. | teenage | "adolescent"[MeSH Terms] OR "adolescent"[All Fields] OR "teenage"[All Fields] |
| 5. | youth | "adolescent"[MeSH Terms] OR "adolescent"[All Fields] OR "youth"[All Fields] |
| 6. | minors | "minors"[MeSH Terms] OR "minors"[All Fields] |
| 7. | people | "persons"[MeSH Terms] OR "persons"[All Fields] OR "people"[All Fields] |
| 8. | population | "population"[MeSH Terms] OR "population"[All Fields] OR "population groups"[MeSH Terms] OR ("population"[All Fields] AND "groups"[All Fields]) OR "population groups"[All Fields] |
| 9. | persons | "persons"[MeSH Terms] OR "persons"[All Fields] |
| 10. | Humans[Mesh] | "humans"[MeSH Terms] |
| 11. | emotional | "emotions"[MeSH Terms] OR "emotions"[All Fields] OR "emotional"[All Fields] |
| 12. | anxiety | "anxiety"[MeSH Terms] OR "anxiety"[All Fields] |
| 13. | depression | "depressive disorder"[MeSH Terms] OR ("depressive"[All Fields] AND "disorder"[All Fields]) OR "depressive disorder"[All Fields] OR "depression"[All Fields] OR "depression"[MeSH Terms] |
| 14. | mental health | "mental health"[MeSH Terms] OR ("mental"[All Fields] AND "health"[All Fields]) OR "mental health"[All Fields] |
| 15. | mental health disorder | "mental disorders"[MeSH Terms] OR ("mental"[All Fields] AND "disorders"[All Fields]) OR "mental disorders"[All Fields] OR ("mental"[All Fields] AND "health"[All Fields] AND "disorder"[All Fields]) OR "mental health disorder"[All Fields] |
| 16. | psychological disorder | "mental disorders"[MeSH Terms] OR ("mental"[All Fields] AND "disorders"[All Fields]) OR "mental disorders"[All Fields] OR ("psychological"[All Fields] AND "disorder"[All Fields]) OR "psychological disorder"[All Fields] |
| 17. | depressive disorder | "depressive disorder"[MeSH Terms] OR ("depressive"[All Fields] AND "disorder"[All Fields]) OR "depressive disorder"[All Fields] |
| 18. | anxiety disorder | "anxiety disorders"[MeSH Terms] OR ("anxiety"[All Fields] AND "disorders"[All Fields]) OR "anxiety disorders"[All Fields] OR ("anxiety"[All Fields] AND "disorder"[All Fields]) OR "anxiety disorder"[All Fields] |
| 19. | affective disorder | "mood disorders"[MeSH Terms] OR ("mood"[All Fields] AND "disorders"[All Fields]) OR "mood disorders"[All Fields] OR ("affective"[All Fields] AND "disorder"[All Fields]) OR "affective disorder"[All Fields] |
| 20. | mood disorder | "mood disorders"[MeSH Terms] OR ("mood"[All Fields] AND "disorders"[All Fields]) OR "mood disorders"[All Fields] OR ("mood"[All Fields] AND "disorder"[All Fields]) OR "mood disorder"[All Fields] |
| 21. | panic disorder | "panic disorder"[MeSH Terms] OR ("panic"[All Fields] AND "disorder"[All Fields]) OR "panic disorder"[All Fields] |
| 22. | bipolar disorder | "bipolar disorder"[MeSH Terms] OR ("bipolar"[All Fields] AND "disorder"[All Fields]) OR "bipolar disorder"[All Fields] |
| 23. | disorder | "disease"[MeSH Terms] OR "disease"[All Fields] OR "disorder"[All Fields] |
| 24. | developing countries[MeSH Terms] | "developing countries"[MeSH Terms] |
| 25. | epidemiological studies | "epidemiologic studies"[MeSH Terms] OR ("epidemiologic"[All Fields] AND "studies"[All Fields]) OR "epidemiologic studies"[All Fields] |
| 26. | prevalence studies | "cross-sectional studies"[MeSH Terms] OR ("cross-sectional"[All Fields] AND "studies"[All Fields]) OR "cross-sectional studies"[All Fields] OR ("prevalence"[All Fields] AND "studies"[All Fields]) OR "prevalence studies"[All Fields] |
| 27. | case-control studies | "case-control studies"[MeSH Terms] OR ("case-control"[All Fields] AND "studies"[All Fields]) OR "case-control studies"[All Fields] OR ("case"[All Fields] AND "control"[All Fields] AND "studies"[All Fields]) OR "case control studies"[All Fields] |
| 28. | cohort studies | "cohort studies"[MeSH Terms] OR ("cohort"[All Fields] AND "studies"[All Fields]) OR "cohort studies"[All Fields] |
| 29. | controlled before- after studies | "controlled before-after studies"[MeSH Terms] OR ("controlled"[All Fields] AND "before-after"[All Fields] AND "studies"[All Fields]) OR "controlled before-after studies"[All Fields] OR ("controlled"[All Fields] AND "after"[All Fields] AND "studies"[All Fields]) OR "controlled before after studies"[All Fields] |
| 30. | cross-sectional studies | "cross-sectional studies"[MeSH Terms] OR ("cross-sectional"[All Fields] AND "studies"[All Fields]) OR "cross-sectional studies"[All Fields] OR ("cross"[All Fields] AND "sectional"[All Fields] AND "studies"[All Fields]) OR "cross sectional studies"[All Fields] |
| 31. | longitudinal studies | "longitudinal studies"[MeSH Terms] OR ("longitudinal"[All Fields] AND "studies"[All Fields]) OR "longitudinal studies"[All Fields] |
| 32. | prospective studies | "prospective studies"[MeSH Terms] OR ("prospective"[All Fields] AND "studies"[All Fields]) OR "prospective studies"[All Fields] |
| 33. | retrospective studies | "retrospective studies"[MeSH Terms] OR ("retrospective"[All Fields] AND "studies"[All Fields]) OR "retrospective studies"[All Fields] |
| 34. |  | **MeSH results: 823** |
